# Creative Flexibility Performance Is Neither Related to Anxiety, Nor to Self-Control Strength, Nor to Their Interaction

**DOI:** 10.3389/fpsyg.2019.01999

**Published:** 2019-08-28

**Authors:** Alex Bertrams, Chris Englert

**Affiliations:** ^1^Department of Educational Psychology, Institute of Educational Science, University of Bern, Bern, Switzerland; ^2^Department of Sport Psychology, Institute of Sport Sciences, Goethe University Frankfurt, Frankfurt, Germany

**Keywords:** anxiety, Bayesian hypothesis testing, creativity, ego depletion, executive functions, creative flexibility, self-control, working memory

## Abstract

Previous research has reliably found that self-control strength moderates the anxiety-performance relationship for cognitive and perceptual-motor tasks that involve executive functioning. In the present preregistered experiment (*N* = 200; https://aspredicted.org/a775h.pdf), we investigated whether the interaction of anxiety and self-control also predicts creative flexibility performance. According to the Attentional Control Theory, anxiety can impair executive functioning. In the case that creative flexibility relies on executive functions, anxiety should therefore interfere with creative flexibility performance. However, self-control strength has been demonstrated to serve as a buffer against the negative effects of anxiety on executive functioning. Therefore, we assumed that there will be a negative relationship between anxiety and creative flexibility performance, and that this negative relationship would be more pronounced for participants who are low compared to high in momentary self-control strength. Analogous to the previous studies, we manipulated the participants’ self-control strength (ego depletion vs. no depletion) and subsequently induced a potentially threatening test situation. The participants then completed a measure of their state anxiety and a standardized test of creative flexibility. Contrary to our expectation, self-control strength, state anxiety, and their interaction did not predict creative flexibility performance. Complementary Bayesian hypothesis testing revealed strong support for the null hypothesis. Therefore, we conclude that, at least under certain conditions, creative flexibility performance may be unrelated to resource-dependent executive functions.

## Introduction

The quality and duration of our lives are oftentimes based on people’s creativity—think, for instance, of the inventions of the light bulb or aseptic techniques. Creativity describes the production of novel and useful products or ideas instead of reproducing previously learned patterns of cognitive responses (Barron, 1955; Mumford, 2003). One important aspect of creativity is *creative flexibility*, which refers to the variety of one’s novel ideas; that is, creative flexibility is reflected by the number of diverging categories under which the meaningful ideas concerning a specific issue can be subsumed rather than the pure amount of novel ideas (Plucker and Kaufman, 2011). Thus, being creatively flexible regarding solutions not only means to leave the usual ways of thinking about something behind, but also to mentally change perspectives repeatedly.

Given the importance of such creative flexibility for human progress, it is highly necessary to understand the preconditions of creativity (Plucker and Kaufman, 2011). Previous research has suggested that creativity is (to some degree) reliant on cognitive abilities (Cropley, 1966), motivation (Collins and Amabile, 1999), or certain personality components (Feist, 1998). However, these variables can only explain a small amount of variance in creative performance, which raises the question of which other processes are behind creativity (Batey and Furnham, 2006). Earlier research viewed creativity as an aspect of intelligence, but today, many researchers agree that although creativity requires a basic level of intelligence (Barron, 1969), creativity and intelligence are not identical constructs (Guilford, 1967; Carroll, 1993). This conclusion is further supported by a recent meta-analysis, that only found a moderate relationship between creativity and intelligence (Kim, 2005).

Some authors have argued that creative performance benefits from effortful, controlled, resource-dependent information processing and executive functioning (e.g., Nusbaum and Silvia, 2011; Benedek et al., 2014). According to this view, to master a creativity task, high attentional control is required. In this context, Nusbaum and Silvia (2011) concluded that “executive cognition is central to creative thought” (p. 36). Executive functions describe basic mental processes that enable us to control our thoughts and behavior (Miyake et al., 2000). The basic executive functions are inhibition, shifting, and updating (Miyake et al., 2000). According to Miyake et al. (2000), inhibition is defined as “the ability to deliberately inhibit dominant automatic or prepotent responses when necessary” (p. 57). Shifting means “shifting back and forth between multiple tasks, operations, or mental sets” (p. 55), while updating enables the “updating and monitoring of working memory representations” (p. 56). These basic executive functions have been found to be relevant for creativity (Vartanian, 2009; Nijstad et al., 2010), because creativity often requires the ability to inhibit certain dominant tendencies, to shift attention to novel cues, and to flexibly change a certain perspective. For instance, brainstorming about novel ways to heal a disease or to advertise a brand requires one to mentally override established concepts and inhibit the individual’s automatic influence on thinking. Simultaneously, one has to direct attention to categories of information that have not yet been considered in this regard, reflect on them, and, when indicated, refuse thinking in this direction and steer one’s attention to the information of other categories. During this cognitive process, one is also mentally engaged by keeping up information concerning important frame conditions. The overarching theoretical framework for these considerations is the controlled-attention theory of creativity (Beaty et al., 2014). According to this approach, “controlled processes provide goal-directed, top-down oversight during creative idea production” (Beaty et al., 2014, p. 1187).

However, there are also other opinions in the ongoing discussion regarding how creative performance is generated. Some authors believe that creativity happens in an effortless, automatic, and resource-independent manner in associative networks and that high attentional control impairs creativity (Wiley and Jarosz, 2012; Furley and Memmert, 2015). Jarosz et al. (2012) even postulate that “superior executive functioning, such as increased attentional control, may in fact be detrimental to reaching creative solutions” (p. 488; see also Stolte et al., 2019). According to this view, high attentional control would hinder one’s creativity because individuals tend to rigidly focus on certain aspects of a given creativity task, which do not lead to a solution while ignoring other, potentially relevant cues (Wiley and Jarosz, 2012). There are also empirical findings, albeit quite a few, that creative flexibility is unrelated to working memory capacity (De Dreu et al., 2012), meaning that controlled attention should not be necessary for creative flexibility.

Creative performance can also be understood as the product of both largely effortless default information processing and executive control processes. Recently, Beaty et al. (2016) have argued in this way and refer in this regard to findings on dynamically cooperating networks of the brain. For our present study, it is crucial that Beaty et al. (2016) assume that creative performance—particularly creative idea generation and evaluation—is also based on executive control.

If creative flexibility depends (partly) on executive functioning, as suggested by the controlled-attention theory (Beaty et al., 2014) and the theory of default and executive control network dynamics (Beaty et al., 2016), it should be hampered by anything that interferes with executive functioning and controlled attention. One influential variable can be anxiety, an emotion that has been extensively studied in a variety of performance contexts (Deese et al., 1953; Hammermeister and Burton, 1995; Zeidner, 1998, 2007; Ashcraft and Kirk, 2001; Biasutti and Concina, 2014). According to the *attentional control theory* (ACT), anxiety impairs executive functioning because anxiety-based worries consume working memory capacity, leading to cognitive interference (Eysenck et al., 2007). Several experimental studies have supported this assumption, showing that anxiety negatively affects inhibition (Calvo, 1996), shifting (Harris and Cumming, 2003), and updating (Calvo et al., 1992) in various tasks. So, if executive functioning is involved in creative performance, higher anxiety should be related to lower creative performance. In line with this notion, a meta-analysis revealed a negative anxiety-creative performance relationship (Byron and Khazanchi, 2011). However, creative flexibility was not explicitly analyzed in this study; therefore, further research is also needed in this respect.

The ACT states that individuals are generally capable of reducing the negative effects of anxiety on executive functioning, but the theory fails to clarify why individuals do not always manage to counteract anxiety-related performance decrements (Englert and Bertrams, 2015). Recent empirical findings hint that the level of momentarily available self-control strength may determine whether anxiety impairs executive functioning and subsequent performance or not (Englert and Bertrams, 2012; Bertrams et al., 2013; Englert et al., 2015). Here, self-control means the process in which an individual volitionally overrides dominant affective, cognitive, or behavioral tendencies (Baumeister et al., 2007).

The *strength model of self-control* states that there is an energy resource on which all self-control acts are based (Muraven and Baumeister, 2000; Baumeister and Vohs, 2016; Baumeister et al., 2018). This resource has a limited capacity, which after having worked on a self-control demanding task, can become temporarily depleted; this depleted state has been named *ego depletion* (Baumeister et al., 1998; Muraven et al., 1998). It has been repeatedly demonstrated that self-control performance suffers during the state of ego depletion (Hagger et al., 2010; Dang et al., 2019). For instance, individuals who had to suppress their emotions while watching an upsetting video clip performed significantly worse in a subsequent self-control task when compared with individuals who did not have to suppress their emotions while watching the identical video clip (Schmeichel et al., 2003). Important for the present study is the finding that attention regulation also requires self-control strength. During the state of ego depletion, individuals are less adept in volitionally controlling their attention (Schmeichel and Baumeister, 2010; Englert et al., 2015).

There is reliable evidence that the effect of anxiety on executive functioning and subsequent performance is moderated by the level of momentarily available self-control strength (Englert and Bertrams, 2015). Individuals only displayed the typical anxiety-related performance decrements in cognitively demanding mental tasks during the state of ego depletion; however, anxious students with intact self-control strength were able to perform at the same level as non-anxious students (Bertrams et al., 2013; Bertrams and Englert, 2014). These findings have also been found in the field of sport psychology: In evaluative, anxiety-provoking test situations, athletes in the state of ego depletion performed significantly worse than their non-depleted counterparts in sports-related tasks that depend on executive functioning (Englert and Bertrams, 2012; Englert et al., 2015). Furthermore, anxious participants’ gaze behavior, which is viewed as a reliable indicator of executive functioning in some perceptual-motor tasks (Henderson, 2003), was less effective in the state of ego depletion, lending further support to the assumption that self-control strength may be the missing link in ACT (Englert et al., 2015). The explanation for these findings is that anxious individuals can in principle use self-control to divert their attention away from anxiety-based worries and focus on the task at hand. In this case there is no cognitive interference and no decline in the executive functioning. However, this compensation of anxiety becomes less effective in the state of ego depletion as self-control decreases (Bertrams et al., 2013, 2016).

The studies that examined self-control strength as a moderator of the anxiety-performance relationship (e.g., Englert and Bertrams, 2012; Bertrams et al., 2013), exclusively used tasks in which either the only correct solution had to be found (e.g., the clearly defined one solution of an IQ task) or a clearly defined 100% mark had to be approached as close as possible (e.g., to be successful in as many of ten basketball free throws as possible). Creative flexibility performance (i.e., generating various solutions) has never been investigated in this regard. The purpose of the present study was therefore to investigate creative flexibility as the dependent measure of performance to supplement the existing studies. In addition, we aimed in this way to contribute to the under-researched relationship between creative flexibility and anxiety as well as executive functioning.

We tested the hypothesis that self-control strength moderates the relationship between anxiety and creative flexibility in the present preregistered study^1^. We adopted the sequential-task paradigm (Baumeister et al., 1998), that is, participants worked on a primary task that either required self-control (depletion condition) or did not require self-control (non-depletion condition). The secondary task was a standardized measure of creative flexibility that had to be solved under evaluative, potentially anxiety-provoking conditions. Moreover, we measured the participants’ anxiety in the evaluative situation. The total procedure paralleled the one used in previous studies that found the moderating effect of self-control strength on the anxiety-performance relationship (Englert and Bertrams, 2012; Bertrams et al., 2013), except for the fact that the secondary task measured creative flexibility performance this time. Analogous to the previous studies, we assumed that there will be a negative relationship between anxiety and performance, and that this negative relationship would be more pronounced for depleted than for non-depleted participants.

## Materials and Methods

### Ethics Statement

The local ethics committee of the Faculty of Human Sciences at the University of Bern approved the present study in advance. Prior to starting the experiment, we obtained written informed consent from all participants. The current study was carried out in accordance with the Declaration of Helsinki and the ethical guidelines for experimental research with human participants as proposed by the Swiss Psychological Society and the American Psychological Association.

### Participants

An *a priori* power analysis using G^∗^Power 3.1 (Faul et al., 2007) indicated that a sample size of at least 160 participants would be required to detect a small effect (power analysis: linear multiple regression, fixed model, *R*^2^ increase, *a priori*; input parameters: *f*^2^ = 0.05, α = 0.05, 1−β = 0.80, number of tested predictors = 1, total number of predictors = 3). We decided to stop data collection either on May 10, 2017 (for organizational reasons) or when we reached a sample size of 210 participants (i.e., 50 people more than the minimum sample size). Most participants were university students recruited in the university buildings. Some participants were recruited by graduate students majoring in educational psychology at the University of Bern who asked their acquaintances to join the study. Among the 210 participants, four (who had mistakenly received the wrong material at some time during the experimental procedure) had to be excluded. Another three were excluded because they suspected the hypothesis (nearly) correctly. One additional participant who did not follow the instructions of the manipulation task was also excluded. These decisions were made prior to data analyses. Because two participants had missing values for the anxiety measure, the final sample consisted of 200 participants (69% female; *M*_*age*_ = 26.67, *SD*_*age*_ = 10.31). The experimenters declared that all participants understood the instructions (all in German) well. All participants gave their informed consent.

### Procedure

Participation took place in a quiet laboratory room with constant lighting conditions in a building at the University of Bern. Experimental sessions were held in groups of up to five participants, each working in a separate, single working partition. First, the participants read and signed the informed consent form.

Next, we applied the sequential-task paradigm. Participants were randomly assigned to either a depletion condition or a non-depletion condition (Baumeister et al., 1998). Therefore, at the beginning of the experiment, we manipulated self-control strength by administering the well-validated transcription task (Bertrams et al., 2010, 2013; Reinhard et al., 2013; Wolff et al., 2013; Dummel and Rummel, 2016; Englert and Bertrams, 2017; Lindner et al., 2017; Wiesner and Lindner, 2017): Announced as an examination of handwriting, all participants transcribed a neutral text about the history of a city on a separate sheet of paper either without using the frequent letters “e” and “n” (depletion condition; *n* = 94) or without omitting any letters (non-depletion condition; *n* = 106). By volitionally overriding well-elaborated writing habits, the participants’ self-control resources should have become temporarily diminished in the depletion condition while remaining relatively intact in the non-depletion condition (Bertrams et al., 2010). An English translation of this task is provided in the supplemental material of a recently published article (Bertrams et al., 2015). After 6 min, the experimenter stopped the participants and asked them to put the material back in an envelope (ensuring that the experimenter remained blind to the experimental condition).

Following the transcription task, the participants worked on a standardized three-item manipulation check (“How effortful did you find the transcription task?”, “How difficult did you find to execute the transcription task?”, and “How much did you suppress your usual writing habits during the transcription task?”; α = 0.80) and completed a single item on perceived competence (“How successful have you been in performing the transcription task?”). All the items had to be answered on Likert-type scales ranging from 1 (*not at all*) to 7 (*very much*). These measures have repeatedly been used in ego depletion research using the transcription task and have found to be reliable (e.g., Bertrams and Englert, 2014; Englert and Bertrams, 2016).

Then, we asked the participants to work on the state version of the Positive and Negative Affect Schedule (PANAS; Watson et al., 1988; Krohne et al., 1996) to rule out the possibility that the transcription task evoked unintended group differences in mood. The PANAS consists of 10 items measuring positive affect (e.g., “happy”; α = 0.87) and 10 items assessing negative affect (e.g., “sad”; α = 0.68). We also included five additional items on subjectively experienced lack of energy (e.g., “inert”; α = 0.82; Abele-Brehm and Brehm, 1986) to explore whether self-perceived exhaustion mediates the effect of self-control strength on the anxiety–performance relationship. The participants indicated on each item how much they were feeling this way at the moment using 5-point Likert-type scales (1 = *not at all* to 5 = *extremely*).

In the next step, we increased the evaluative character of the testing situation by adapting the instructions from previous anxiety research (Murray and Janelle, 2003; Behan and Wilson, 2008; Bertrams et al., 2013). The participants received a sheet that announced a test on personal creativity, explained the enormous importance of creative flexibility, and informed them that they would receive personal face-to-face feedback about their performance and that their performance would be compared with the performance of other participants. Moreover, the participants were informed about the time limit of 2.5 min. After signaling that they had carefully read the instructions, they received the original instructions of the subtest *Features-Abilities* (Eigenschaften-Fähigkeiten) from the Berlin Intelligence Structure Test (Berliner Intelligenzstruktur-Test; BIS), which included a sample item (Jäger et al., 1997). Previous research has revealed that announcing a test this way elicits differences in state anxiety between participants (Bertrams et al., 2013).

Afterwards, we administered the well-validated brief version of the State-Trait Anxiety Inventory (STAI-SKD) to measure the participants’ level of state anxiety (Englert et al., 2011). Five items (e.g., “I am nervous”) had to be answered on 4-point Likert-type scales, which indicated how they felt at that specific moment (1 = *not at all* to 4 = *very*, α = 0.83). The STAI-SKD has been demonstrated to be sensitive to situational threats induced by evaluative performance situations (Bertrams and Englert, 2013). For such situations, it has also been shown that the STAI-SKD captures variance in state anxiety stemming from individual differences in trait test anxiety (i.e., susceptibility to become anxious when facing a test) (Bertrams and Englert, 2013).

Then, it followed the performance measure of creative flexibility. As previously mentioned, creativity can be understood as a process in which an individual is generating novel ideas (Barron, 1969; Mumford, 2003). For this reason, we measured creativity by administering a standardized test, specifically, the subtest *Features-Abilities* (Eigenschaften-Fähigkeiten) from the BIS (Jäger et al., 1997). Specific subtests of the BIS have repeatedly been applied in previous creativity research, including to measure creative performance as an independent variable in experiments (Benedek et al., 2012; Lichtenfeld et al., 2012; Roeser et al., 2015). The participants were asked to write on the response sheet as many different features and abilities that a seller should *not* have as possible. According to the manual, after 2.5 min, the experimenter stopped the task. Creative flexibility was coded according to the standardized guidelines in the test manual (i.e., counting the number of various categories in a participant’s responses) once by a group of inexperienced educational psychology students and then independently by an experienced research assistant. Because both codings were highly correlated (*r* = 0.69, *p* < 0.001), we took the mean of both as our dependent variable of creative flexibility (note that the results did not change by only using either coding).

After the measure of creative performance, the participants completed a post-experimental questionnaire. In the questionnaire, the participants’ knowledge of the actual hypothesis was checked, and information about gender, age, education/employment, and language ability was requested. Finally, the participants were thanked and carefully debriefed.

## Results

### Preliminary Analyses

We conducted *t*-tests to check the success of the experimental manipulation. For this, we applied two-tailed testing against a 0.05 level of significance. The manipulation check indicated that our manipulation of self-control strength was successful because the depletion group (*M* = 3.83, *SD* = 1.30) gave a higher rating than the non-depletion group (*M* = 3.10, *SD* = 1.35), *t*(198) = 3.88, 95% CI of the difference [0.36, 1.10], *p* < 0.001, *d* = 0.55. There was no statistically significant difference between the depletion and non-depletion groups in how successful the participants perceived themselves in the transcription task, *p* = 0.16. Moreover, the transcription task did not result in differences between the two experimental conditions in positive affect or negative affect, *p*s > 0.06. Thus, the transcription did not produce unintended differences in self-confidence or mood (still, we controlled for these variables in the auxiliary analyses; see below). As in previous research (Bertrams et al., 2013), the two experimental groups also did not differ in state anxiety, *p* = 0.36. The *SD*s of state anxiety in the depletion (*M* = 1.76, *SD* = 0.53) and the non-depletion condition (*M* = 1.69, *SD* = 0.49) resembled the ones in two previous experiments that revealed a significant interaction between ego depletion and state anxiety in regressing performance (*SD* interval in Bertrams et al., 2013: [0.46, 0.56]).

### Main Analyses

The mean for creative flexibility (number of various categories) was 7.03 (*SD* = 1.87) for the overall sample, 7.07 (*SD* = 1.66) for the depletion condition, and 6.99 (*SD* = 2.04) for the non-depletion condition. Neither self-control strength (experimental condition) nor state anxiety correlated with creative flexibility, *r*(198) = −0.02, 95% CI [−0.16, 0.12], *p* = 0.77, and *r*(198) = 0.02, 95% CI [−0.12, 0.16], *p* = 0.80, respectively (bivariate correlations, two-tailed tests). Applying a hierarchical multiple regression analysis, we regressed creative flexibility on the manipulation of self-control strength and *z*-standardized state anxiety scores, adding their interaction in a second block. For each predictor, we tested against the significance level of α = 0.05 (two-tailed tests). As can be seen in Table 1, none of the independent variables significantly predicted creative flexibility (*p*s > 0.44). The regression slopes were small and even contrarily directed to what a resource-dependence view of creativity would suggest.

**TABLE 1:** Hierarchical multiple regression analysis regressing creative flexibility on self-control strength (experimental condition), state anxiety, and their interaction.

**Block and predictor**	**Main-effects model**	**Full model with interaction**
**Block 1: main effects**		
Self-control strength^a^	*B* = −0.07, 95% CI [−0.60, 0.45], β = −0.02, *p* = 0.78	*B* = −0.07, 95% CI [−0.60, 0.45], β = −0.02, *p* = 0.78
Anxiety	*B* = 0.03, 95% CI [−0.23, 0.30], β = 0.02, *p* = 0.81	*B* = 0.13, 95% CI [−0.24, 0.50], β = 0.07, *p* = 0.48
**Block 2: interaction**		
Self-control strength × anxiety		*B* = −0.21, 95% CI [−0.73, 0.32], β = −0.08, *p* = 0.44
Overall model *R*^2^	0.001	0.004
Adjusted *R*^2^	−0.009	−0.01
Δ*R*^2^	0.001	0.003
Δ*F*	0.07 (*p* = 0.93)	0.59 (*p* = 0.44)
Overall *F*	0.07 (*p* = 0.93)	0.25 (*p* = 0.87)
*df* for overall *F*	2, 197	3, 196

**N* = 200. ^*a*^Coding of the experimental conditions: 0 = depletion, 1 = no depletion.*

We checked for univariate outliers (*z*-standardized state anxiety scores > 3.29) and for multivariate outliers (Mahalanobis distance values > 16.27), as recommended by Tabachnick and Fidell (2007). In this way, we detected one outlying case; however, repeating the regression analysis while excluding this participant did not change the results. Inspecting the normal probability plot of the regression standardized residual led us to conclude that no serious violation of the normality assumption was given. Normalizing the slightly skewed state anxiety scores by applying a log transformation (Tabachnick and Fidell, 2007) and repeating the regression analysis did not alter the results. Furthermore, when entering positive affect, negative affect, self-reported lack of energy, and perceived competence during the transcription task as covariates, the results remained the same. Because we did not find the predicted effect in the main analyses, we omitted the preregistered analysis on whether an experienced lack of energy mediates it.

### Complementary Bayesian Hypothesis Testing

Given the null results reported in the main analyses, we aimed to conclude that the assumed relationship between anxiety and creative flexibility under ego depletion does not exist (i.e., that the null hypothesis is likely to be true). In contrast to conventional null hypothesis statistical testing (NHST), Bayesian hypothesis testing allows for the quantification of evidence for the null hypothesis (Dienes, 2014; Wagenmakers et al., 2018a, b). Therefore, we applied the functions Bayesian Correlation Pairs and Bayesian Linear Regression in the software JASP (JASP Team, 2018) to determine the relevant Bayes factors that could express the intensity of the evidence that the data provide for the null hypothesis versus the alternative hypothesis (BF_01_). To interpret each BF_01_, we used the classification scheme provided by Wagenmakers et al. (2018a).

There was “strong evidence” that there was no bivariate correlation between self-control strength (experimental condition) and creative flexibility, BF_01_ = 14.08 [settings: correlated positively (i.e., alternative hypothesis = creative flexibility performance is lower in the depletion compared with the non-depletion condition), stretched beta prior width = 1]. Moreover, the evidence was “strong” that state anxiety did not correlate with creative flexibility, BF_01_ = 13.72 (settings: correlated negatively, stretched beta prior width = 1). These results indicate that the observed data are 14.08 times and 13.72 times, respectively, more likely under the null hypothesis (i.e., no correlation exists) than under the alternative hypothesis (i.e., there is a correlation).

Next, we regressed creative flexibility on the experimental manipulation of self-control strength, *z*-standardized state anxiety scores, and their interaction. The multiple regression analysis revealed “very strong evidence” that the full model (i.e., the two main effects plus their interaction) did not predict creative flexibility, BF_01_ = 69.67. Furthermore, the evidence was “strong” that the main-effects model was also not predictive of creative flexibility, BF_01_ = 26.08. In other words, the null model outpredicted the two models that contained the predictors.

## Discussion

Previous research has suggested that the negative effects of anxiety on certain types of performance can be reduced by engaging in self-control. Therefore, if one’s self-control resources are momentarily depleted, higher anxiety should be related to weaker performance (Englert and Bertrams, 2015). This assumption has been empirically supported in several studies, which have shown that ego depletion moderates the effects of anxiety on performance in different cognitive (e.g., Bertrams et al., 2013) and perceptual-motor tasks (e.g., Englert and Bertrams, 2012) that depend on executive functioning. However, this has not yet been tested for performance in creative flexibility. The present study addressed this shortcoming: Paralleling the procedure of previous studies, we examined whether anxious participants would be less creatively flexible when their self-control strength is depleted when compared with non-depleted. However, we found that reduced self-control strength, anxiety, and their interaction did not predict performance in creative flexibility. This null finding stems from a sufficiently large sample size of *N* = 200 (note that the present sample was large enough to detect a relatively small effect of *f*^2^ = 0.04). In addition, Bayesian hypothesis testing revealed strong evidence for the null hypothesis (i.e., there is no relationship between the assumed predictors and creative flexibility).

A potential explanation for the unexpected pattern of results may lie in our general assumption that creative flexibility is based on executive functions. As noted in the introduction, there is also a view in the creativity literature that creative performance does not depend on executive functioning. Instead, it is assumed that the creative process takes place in associative networks and is effortless, automatic, and resource-independent (Wiley and Jarosz, 2012; Furley and Memmert, 2015). In line with this notion, De Dreu et al. (2012) found creative flexibility to be unrelated to working memory capacity. Our results may contribute to the debate on whether creativity is resource dependent or, at least under certain conditions, primarily an automatically executed process. Optimistically, one could say that the important human ability to creatively come up with various novel ideas is not easily impaired by adverse conditions and negative affect. Future experimental research designs may offer further insight about this observation. Thereby, one should keep in mind that the present study has only examined a very specific type of creative performance and is not necessarily meaningful for all creative tasks (e.g., the composition of a witty piece of music).

Another explanation would be that the methods in the present study did not work or even that ego depletion just does not exist (Carter et al., 2015; but for a contrary perspective, see also Baumeister et al., 2018). We do not want to go into a theoretical in-depth discussion about the existence or non-existence of ego depletion here. Instead, we would like to point out that we used established procedures and measures that have been repeatedly and successfully applied in previous research to investigate whether the interaction between self-control strength and anxiety predicts performance (Englert and Bertrams, 2012, 2015; Bertrams et al., 2013; Englert et al., 2015). Using these methods has reliably revealed statistically significant results across a wide range of performance domains (e.g., reasoning, mental calculation, dart tossing) and groups of participants (e.g., athletes, school students, undergraduates). Therefore, we favor the above mentioned explanation that, under specific conditions, creative flexibility does not depend on executive functioning. This interpretation is supported by the fact that both ego depletion and state anxiety did not predict creative performance in the present study, and overall, the adverse effect of anxiety on executive functions is well established (Eysenck et al., 2007).

Taken together, the present study did not support our main hypothesis that ego depletion moderates the effect of anxiety on creative flexibility. Moreover, there was no main effect of ego depletion or state anxiety. Nonetheless, our findings can add to the discussion on how creative behavior is generated, suggesting that at least sometimes creative flexibility seems to be independent of executive functions (cf. De Dreu et al., 2012). Bayesian hypothesis testing (Wagenmakers et al., 2018a, b) may be an appropriate way to test the diverging assumptions from different theoretical accounts in creativity research (requiring vs. not requiring executive functions) against each other. With regard to our research program on the moderation of the anxiety-performance relationship through self-control, we must limit the scope of validity: Creative flexibility does not seem to be predictable in the same way as other types of performance we have studied in the past (e.g., Englert and Bertrams, 2012; Bertrams et al., 2013).

However, the findings of the present study must not be overgeneralized. Creativity is a rather broad construct, which means that various types of performances with their specific measurements can be subsumed under this term (Villani and Antonietti, 2013). It is still possible that other measures of creative performance such as the *draw an alien task* (Ward, 1994) would depend on anxiety and self-control strength. The same may be the case when other techniques to evaluate creativity are used, for example, the newly developed metric *forward flow* (Gray et al., 2019). Furthermore, certain task parameters of the creativity measure (e.g., the task duration; see Beaty and Silvia, 2012) could influence the results. In addition, sophisticated statistical methods that can account for variability in relevant person-related factors (e.g., the personality dimension openness to experience or the momentary mood) may be applied. For instance, linear mixed-effects models (Barr, 2013) which allow to control for such variability may yield a different finding than the present one. Our study may thus be considered as only the first step in investigating the relational pattern of anxiety, self-control strength, and creativity, and can serve as a reference point for corresponding future research.

## Data Availability

The datasets generated for this study are available on request to the corresponding author.

## Ethics Statement

This study was carried out in accordance with the recommendations of the Swiss Psychological Society and the American Psychological Association with written informed consent from all subjects. All subjects gave written informed consent in accordance with the Declaration of Helsinki. The protocol was approved by the ethics committee of the Faculty of Human Sciences at the University of Bern.

## Author Contributions

AB and CE contributed meaningfully to the manuscript, developed the study concept and design, and approved the final version to be published and agreed to be accountable for all aspects of the work in ensuring that questions related to the accuracy or integrity of any part of the work are appropriately investigated and resolved. AB analyzed and interpreted the data and prepared the draft manuscript. CE provided the critical revisions.

## Conflict of Interest Statement

The authors declare that the research was conducted in the absence of any commercial or financial relationships that could be construed as a potential conflict of interest.

## Footnotes

1 https://aspredicted.org/a775h.pdf
